# Vaccinations and Vaccinators’ Tracking System in Island Settlements: CHAD 2017-2018

**DOI:** 10.29245/2578-3009/2021/S2.1116

**Published:** 2021-04-12

**Authors:** Ajiri Atagbaza, Joseph Okeibunor, Felix Amadou, Souley Kalilou, Aime Matela Esanga, Adama Nanko Bagayoko, Philbert Bohoussou, Obianuju Igweonu, Mahamat Mbodou Seid, Ahmad Jibril Aliyu, Elizabeth Benoit Ntezayabo, Mohamed Alimou Traore, Mwanza Nzioki, Adebola Olaleye, Adele Daleke Lisi Aluma, Djibrine Abakar Sedick, Adam Mahamat Seid, Mahamat Saleh Tahir, Narcisse de Medeiros, Bakoly Rabenarivo, Fabien Diomande, Pascal Mkanda

**Affiliations:** 1 WHO Regional Office for African (WHO AFRO), Brazzaville, Congo; 2 WHO Consultant Lake Chad; 3 Ministry of Health, Chad; 4 University of Nigeria, Nsukka; 5 WHO, Chad; 6 WHO Headquarters, Geneva; 7 Independent Consultant; 8 UNICEF Dakar; 9 UNICEF, Cameroon; 10 CDC Atlanta

**Keywords:** Tracking vaccination teams, Dashboard, Team performance, Geographic coverage of settlements

## Abstract

**Introduction:**

Chad is a country within the Lake Chad sub region, currently at risk for poliovirus infection. The Lake Chad Task Team on polio eradication in this sub region made significant efforts to reduce the risk of polio transmission in Chad by tacking immunization teams in the Island Settlement using a Geographic Information System (GIS) technology. This article demonstrates the application of GIS technology to track vaccination teams to monitor immunization coverage in the Island settlements, reduce the number of missed settlements, to provide evidence for vaccination implementation and accountability and improve team performance.

**Methods:**

In each district where tracking was conducted, global positioning system–enabled Android phones were given to each team on a daily basis and were used to record team tracks. These tracks were uploaded to a dashboard to show the level of coverage and identify areas missed by the teams.

**Results:**

In 2018, tracking covered 30 immunization days, in six rounds. Approximately average of 1173 Island settlements were tracked and covered in each of the six rounds. A total of 806,999 persons aged 0-10 years were immunized, out of which 4273 were zero dose cases at the point of their immunization. Tracking activities were conducted. There was an improvement in the geographic coverage of settlements and an overall reduction in the number of missed settlements.

**Conclusions:**

The tracking of vaccination teams and Island settlements ensured useful information for planning and implementation of polio campaigns and enabled supervisors to evaluate performance of vaccination teams

## Introduction

Poliomyelitis is a highly infectious disease caused by polioviruses and is among the causes of irreversible paralysis in children and adults^1^. The clinical manifestation is paralysis which occurs a few hours or days after contracting the virus. Most polio cases are asymptomatic, which helps to sustain its transmission through faeco-oral; route, and communities; with poor hygiene and sanitation are at high risk of virus transmission^2,3^. Immunization against polio virus was one of the key strategies for interrupting transmission and eradicating polio in the endemic countries.

Immunization against vaccine-preventable diseases l health status in the African Region. It has contributed to the successful smallpox eradication and has the potential for reducing the incidence, morbidity and mortality due to most other vaccine preventable diseases^4–9^. The recent reduction of measles death by 84% between 2000 and 2011 is also linked with the successful implementation of immunization programs in the Region. Following global, regional, and national efforts in the promotion of immunization, DPT3 coverage, a generally acknowledged indicator of the performance of immunization programs, rose from 5% in 1974 to 74% in 2010 in the African region^23^. The polio scourge was actually brought under control as many previously endemic countries became polio-free due to large scale immunization with the poliovirus vaccine^10^.

Between 2000 and 2012, the number of African countries endemic with the wild poliovirus (WPV) decreased from 12 to one and new WPV cases reported in 2012 decreased significantly by 63%, with 128 cases in only three countries (Nigeria, Chad, and Niger) compared to 350 cases in 12 countries in 2011^11^. By July 2014 only six polioviruses were reported in the African Region, and these were in Nigeria^12,13^. However, two years after there was a detection of an ongoing outbreak of type 1 wild poliovirus (WPV1) in Nigeria. That was followed by four cases of WPV1 that have been reported in Borno State, Nigeria in July 2016, exactly two years since the last WPV1 cases were reported in the Region.

Central Africa thus once again became faced with the threat of poliovirus. Detection of wild poliovirus type 1 (WPV1) in 2016 in north-eastern Nigeria meant the entire region is currently at risk. Chad, along with other countries of the Lake Chad sub-region, declared this outbreak to be a regional public health emergency and is implementing a regional outbreak response, coordinated with neighbouring countries^24^. The four key strategies of the Global Polio Eradication Initiative (GPEI) namely, high coverage with oral polio vaccine in routine immunization, surveillance for acute flaccid paralysis, supplemental immunization activities (SIAs), and mop-up immunizations, were activated in responding to the Nigerian outbreak within the lake Chad sub-region, including the Republic of Chad^25^. The sub-region, and indeed Chad remained at risk for wild poliovirus (WPV) as a result of several factors but mainly because of poor quality and coverage of the SIAs. Another hindrance was the inability to identify areas that were missed by timely intervention with vaccination to provide the required herd immunity to break the polio transmission^14^. Immunization coverage has been sub- optimal due to many factors including vaccinators issues.

The Lake Chad Task Team for polio eradication coordination within the sub-region deployed geographic information system (GIS) technology to track vaccination teams during polio campaigns, particularly in the Island settlements in Chad. These innovations are now increasingly used by public health professionals to visualize and explore disease patterns1^4,18^. Similar tools were also used in India to monitor population migration through railroads, and Google Earth was used to track the poliovirus down the Congo River^14^ demonstrating the critical role of GIS as a tool for making informed decisions on health, social, and environmental issues. Since the deployment of this technology, immunization coverage has increased significantly in the Island Settlements of Chad, which were previously unvaccinated. However, this has not been covered in the literature. This paper documents the deployment of the GIs tracking system in tracking vaccination teams during SIAs in the Island settlements in Chad.

## Methods

### Study Area and Population

The geographical area for the island vaccination campaign was eight health districts in Lac and Hadjer- Lamis regions in Chad (Figure 2) at high risk for polio transmission where the island vaccination campaign tracking exercise was conducted. These health districts contained forty-nine health areas, with an estimate of over one thousand five hundred (1,500+) island settlements. The 2018 projected population in these districts was 859,887 people.

### Data Collection and Mapping

eHealth Africa worked in collaboration with the Lake Chad Task Team, MoH, and WHO in Chad to implement a Vaccinator Tracking System (VTS) project in eight districts for the polio vaccination campaign. Each eHealth Africa Project Field Officer (PFO) was assigned to districts and subsequently supervised the in-country trained consultants in their respective districts as well. They brought all necessary supplies to the field and were present during the campaign to provide training and technical support. In the respective districts, the Health Center Focal Persons subsequently trained the district vaccination teams and oversaw deployment to the field.

## Supplies

eHealth Africa provided the following supplies for the field activity to be returned upon project

**completion**
- Four hundred mobile phones (Huawei Gaga)
- Four hundred solar chargers (Creative Solar Charger CTE-6087S)
- Two charging boards (Tower Extension Set SU-T40 USB)
- Two IDP laptops (Lenovo Thinkpad T540PL)
- Two mobile printers (HP Officejet 100 Mobile Printer)
- Two extension cords
- Other: box locks, masking tape, markers, A4 printer paper, printer cartridge

The Lake Chad Task Team contributed the following supplies, to be returned upon project

**completion**
- Two projectors
- Two generators

All supplies were left at the WHO office in N’Djamena to be used for subsequent campaign tracking. eHealth Africa worked in collaboration with the Lake Chad Task Team, MoH, and WHO in Chad to implement a Vaccinator Tracking System (VTS) project in the eight health districts of Bol, Bagasola, Liwa, Ngouri, Kouloudia, Isseirom, Mani and Karal for the polio vaccination campaign which took place from November 20 to November 24, 2018. Two Project Field Officer (PFO) eHealth Africa were sent to Chad to support the in-country team. They brought all necessary supplies to the field and were present during the campaign to provide training and technical support. Trained Health Center Focal Persons subsequently trained the health district vaccination teams and oversaw deployment to the field.

The Health Center Focal Persons distributed the phones at the beginning of the campaign. Due to distance and poor network reliability, the campaign did not allow for daily collection/distribution. Instead the vaccination team used solar chargers to ensure that the phone was always charged. Vaccinators carried the phones during the campaign and when they stopped to vaccinate for two minutes, the device recorded a GPS track. The two minutes’ time measurement is based on the average time a vaccinator spends vaccinating in one place. Metrics have been collected and optimized over time throughout all VTS projects to ensure accuracy of this time limit). During the campaign, the focal persons worked hand in hand with the PFO to ensure that all devices were functioning properly.

At the end of the campaign, phones were retrieved and the information collected on the devices was synced by the PFOs. Stakeholders were able to see where each vaccination team covered through map visualization and a coverage percentage via the shared dashboard. During each of the campaign, the VTS support team comprising of two EHA PFO’s, and six in-country trained consultants are deployed to all of the eight island districts.

## Training on Tracking Vaccination Teams

**Figure 1:**
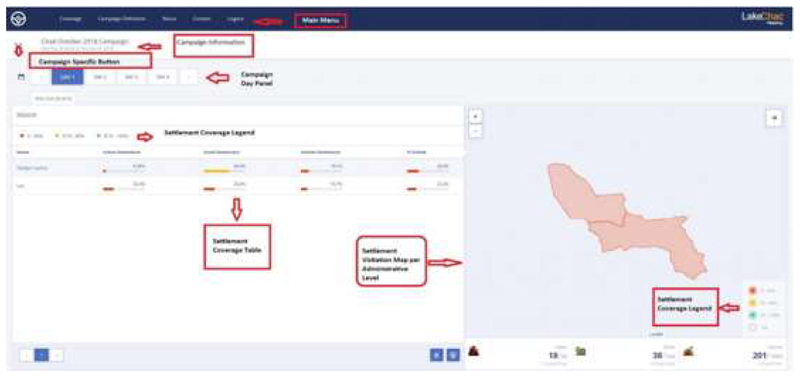
Vaccination Tracking System (VTS) Dashboard

To track vaccination teams during immunization campaigns and visualize settlement coverage, a vaccination tracking system (VTS) dashboard was developed^14^. See Figure 1. In the VTS dashboard, settlements were categorized into large and small settlement areas. The training preceding field activities is held on one day at the District Health Center. A typical training center has in attendance was theMedecin Chef de District (MCD), Chef de Zone, RPEVs, Consultants, Health Center Focal Persons, and Assistants. All responsible persons were trained on project expectations and device usage.

## Vaccination Tracking Process

The tracking exercise was implemented in 49 health areas in eight high-risk island health districts in Chad after the subsequent exercises which were intended to monitor the progress of vaccination tracking activities within the Lake Chad Basin. Mani and Bol were initially selected as the pilot health districts due to a high number of Islands within those health districts. The existence of numerous islands within Bol and Mani has in the past impaired vaccination activities, hence the need to monitor and track the activities there. Starting from the April campaign, the selected island health districts have since been increased to eight health districts namely: Bol, Bagasola, Liwa, Ngouri, Kouloudia, Isseirom, Mani, and Karal.

**Figure 2:**
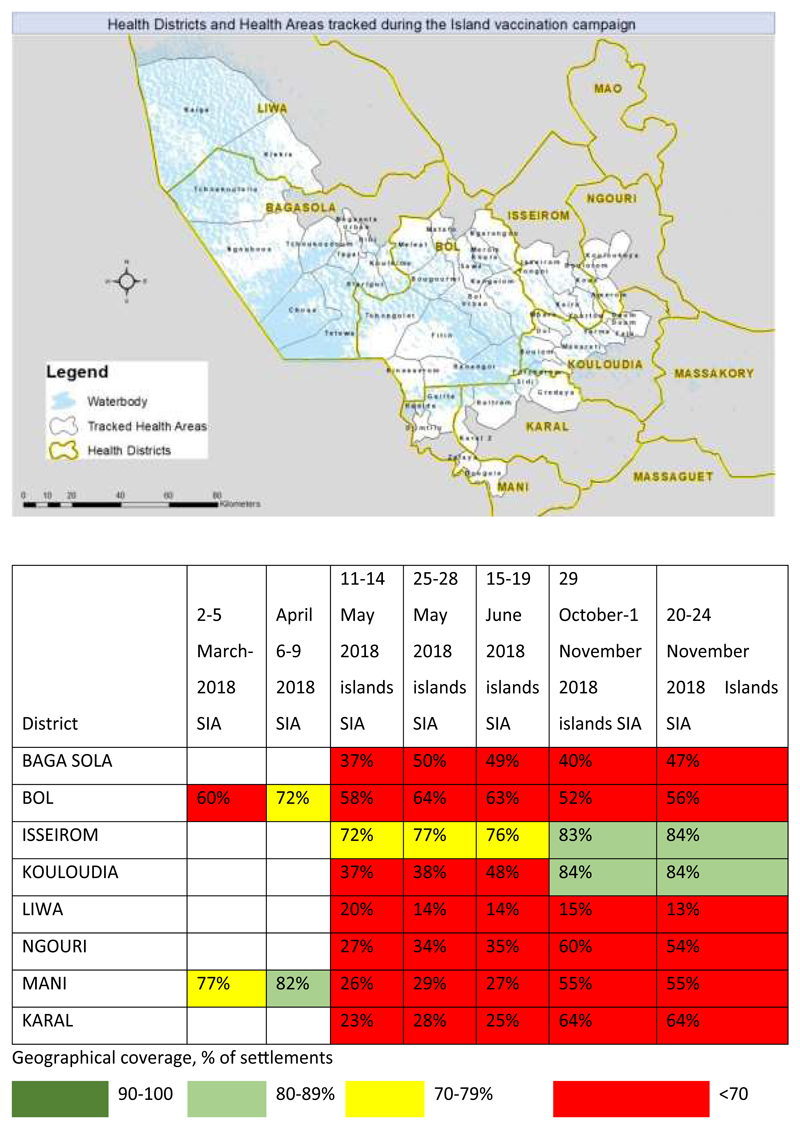
Islands tracked, by campaign month between 2017 and 2018 mapped by geographic information system technology.

**Figure 3:**
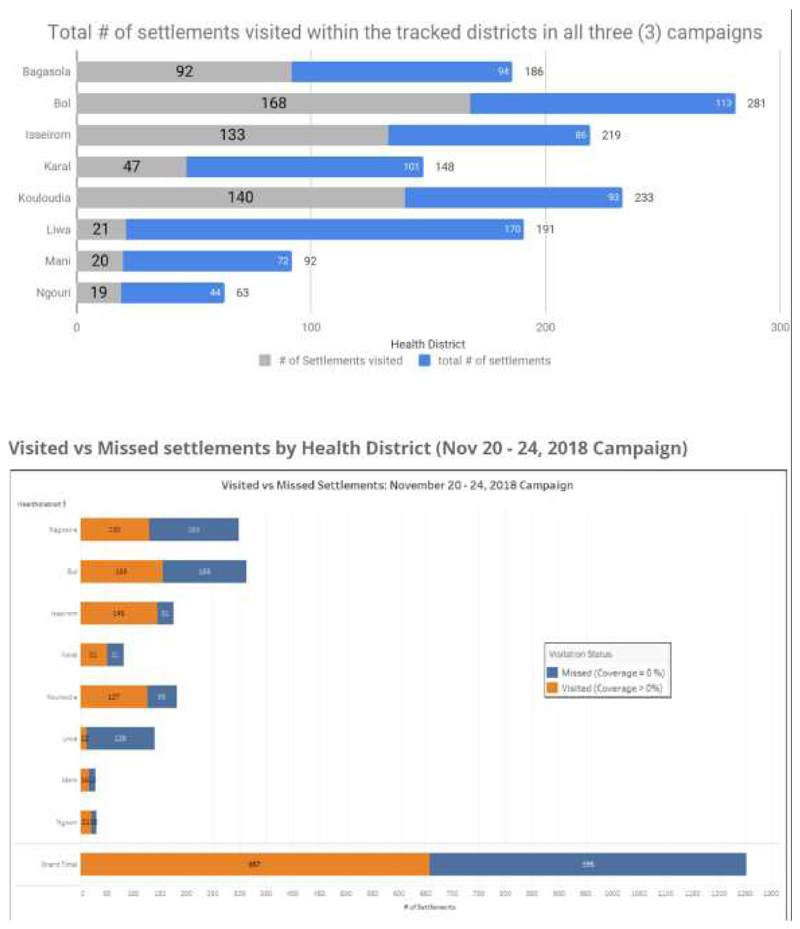
Island Settlement Areas visited by vaccination teams during polio campaigns in Chad, highlighting main vaccination activities (days 1–3 of campaigns) and mop-up activities (day 4; final campaign day)

Furthermore, a missed or poorly covered settlement report was generated at the end of day four of the immunization campaign and shared with the field team. The partially covered and missed settlement reports showed all settlements in which the cumulative percentage of areas visited was below a certain threshold. A settlement was considered partially covered if <70%, 50%, and 100% of the urban, hamlet, and small-settlement areas, respectively, were covered with tracks. On the other hand, urban, hamlet, and smallsettlement areas with coverage of <50%, <40%, and <1%, respectively, were classified as missed^14^. At the end of day five of the campaign, the missed settlement report was validated at three different levels to ensure that the final post campaign list included only settlements not visited by vaccination teams. An automated vaccination tracking report after the campaign was also generated. The identified missed areas were then visited for a focused vaccination. See Figures 2 and 3 for the settlements tracked.

## Estimation of Geographic Coverage

To estimate the geographic coverage for all the settlement types, GPS positions were compared to positions at the smallest denominators. The geographic coverage data were analyzed to show two key indicators: (1) the cumulative percentage visited (for the various settlement), defined as the percentage of smallest denominators (ie, grid squares for urban areas or buffered points for small settlements) that have intersected at least once with a team’s GPS tracks; and (2) the total percentage visited, calculated as the average cumulative percentage visited across all settlements.

GPS positions were collected every two minutes, but only GPS tracks that satisfied the following rules were considered valid: for urban and small-settlement areas, tracks were made within the campaign days, were made between 5 am and 6 pm, and had a speed at the capture time of <1 m/second; and for hamlet areas, tracks were made within the campaign days and between 5 am and 6 pm.

The settlement coverage and visitation status are ascertained when a settlement shows evidence of ‘visit’ by intersecting a valid GPS track. As part of the process for deploying the Chad VTS, GPS enabled devices are handed to the field enumerators (or vaccinators as in the case of the Polio Vaccination Tracking campaigns) to track the locations of settlements they visited. These phones have apps installed that generate passive GPS tracks every 90 seconds and these tracks are separated into valid and invalid tracks based on whether the vaccinator operated at a speed less than 2m^2^ (valid track) or greater than 2m^2^ invalid track suggesting that at such speed a vaccination could not have been said to take place). Where a valid track intersects the unit for coverage calculation. For example, in a built-up area 50x50m of grids, the settlement is said to be visited (not missed), and the percentage coverage is calculated based on the number grids visited relative to the total.

The lowest unit for calculating Geographic coverage on the Chad VTS is the Settlement (referring to the Hamlet, Small settlement, and Built-up areas). Settlement coverage is further aggregated at the ward/health area, local government/district, and state/region administrative levels as may be required. Hamlet Areas’ Coverage: Hamlet areas are 200m buffers generated around Hamlet points denoting the location of hamlet settlement types. These 200m buffer polygons are dissolved where they intersect other adjoining hamlets that are within 200m and also bear the primary name of the settlement or location and in some cases a machine named settlement where no settlement name exists for that settlement.

For coverage calculation, 50m buffers are generated around the individual Hamlet settlement points within a Hamlet Area. The 50m hamlet buffers maintain a unique identifier that ties it to the respective Hamlet area it falls within, and the geographic coverage of the hamlet area is usually the percentage of visited ‘50m’ hamlet buffers within the hamlet area; calculated as total visited 50m hamlet buffer/total 50m hamlet buffers within the Hamlet area multiplied by 100 (percent).

Built-up area Coverage: In calculating the built-up area (BUA) settlement coverage 50x50m grids representing regular sections of the BUA settlement polygon are generated within the BUA. Before generating the 50x50m grids, areas of the settlement representing bare ground or non-residential areas are as much as possible cropped out or excluded from the polygon area. This is to ensure that all grids are representative of actual parts of the settlement assessed. Each grid within the built-up area maintains a unique identifier (or id) that relates it with the parent built-up area.

Small Settlement Coverage: Small settlements are identified with a point feature representing the location of the small settlements and a 75m buffer that reflects the name of the settlement and also serves as a basis for computing geographic coverage. A small settlement is said to be visited and 100% covered if it intersects a valid anywhere within the 75m buffer.

Aggregating Coverage at the administrative levels

The calculated coverage at the health area and district levels is the average percentage coverage of all visited settlements calculated distinctly for each settlement type e.g. Built-up area, Small settlement area, and Hamlet areas. For instance, if the average coverage of all Built-up areas within a Health area is X%, Y% for Small settlements, and Z% for Hamlet areas, the overall coverage for the Health area will be the sum of X%, Y%, and Z% divided by three as shown in the screenshot of the dashboard.

## Vaccination Reach

VTS tracks can be used to map the vaccination reach, by number of contacts, using up to date settlement information. For the purposes of this study, we defined the terms reached and unreached as vaccination status classifications that could be applied to settlements and their estimated populations. The classification reached indicates settlements that have GPS evidence of at least one visit by tracked vaccination teams during working hours of a vaccination campaign or intervention since the start of the response or over a specified period. Unreached is the classification for settlements which do not have evidence of visitation by vaccination teams, but still show evidence of habitation. Together this binary classification scheme of reached and unreached has been termed vaccination reach^22^.

Nigeria and Chad were independently assessed for vaccination reached based on campaign independently carried out between August 2016 and October 2018. Whilst the emphasis was placed on Chad Islands, Borno State in Nigeria was the target location with special activities like Reaching Inaccessible Children (RIC), Reach every settlement (RES), and House to House (H2H) also taken into account.

## Results

A database comprising of 10,500+ settlements and over 16,000 Points of Interest (Pols) were collected between June 2017 and March 2018 in Chad. This was used to support five tracking campaigns from March to November 2018. The data was collected, analyzed, and provided to support systematic planning and accountability for vaccination campaigns via the Field Tracking System (FTS), which showed that the island settlements in Chad were being effectively and incrementally reached during vaccination campaigns (Figure 5). While the tracking dashboard does not give us the coverage of the number of children vaccinated, it provides essential and historical information tracking daily activities for each campaign day. Users are able to identify which areas were visited and understand the coverage at the settlement, health area and health district levels. It serves as a monitoring and accountability tool^21^ ensuring that vaccinators make actual visits and missed settlements easily identified.

**Figure 4:**
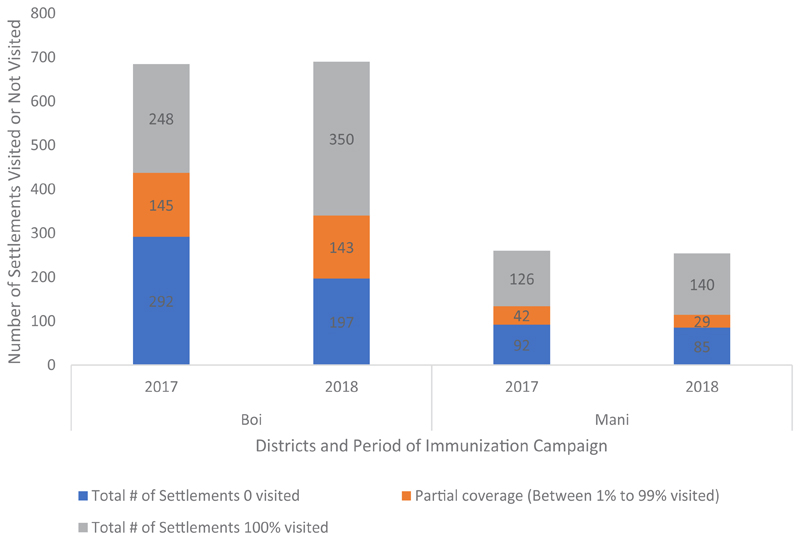
Proportion of settlements in Chad islands visited over time: 2017-2018 2^nd^-5 March 2018 Chad SIA

**Figure 5:**
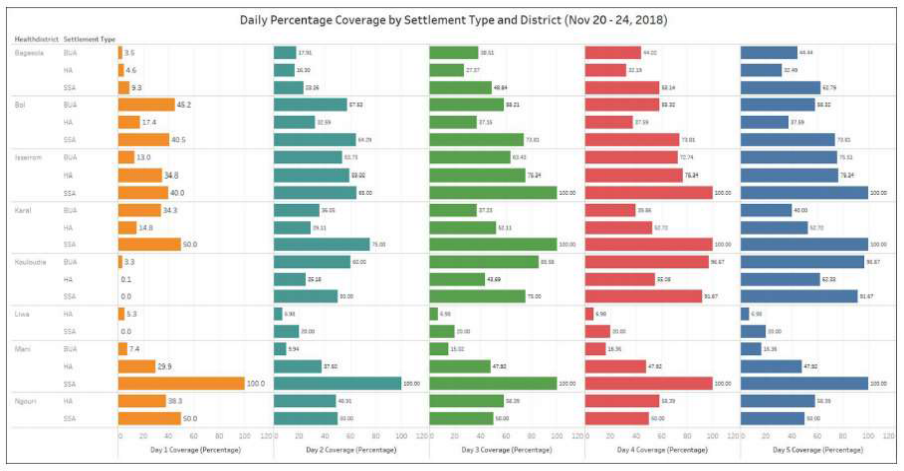
Day-by-day settlement coverage comparison per Settlement Type and health district (November 20-24, 2018 Campaign)

Data analysts across Chad, Cameroun & Niger were trained within six training sessions to enable geospatial planning for field activities. Two locally hired and trained team members have been absorbed into the WHO Chad and AVADAR (Niger) project to support geospatial analysis and field planning and they’re using geospatial knowledge acquired in the course of the project. Figure 4 shows the proportions of settlements visited and those not visited between the immunization campaigns of 2017 and 2018 when the GIS technology was deployed for tracking vaccinators. It showed general improvement in the proportion of settlements visited while the proportion of unvisited settlements shrunk with time. For instance, in 2017 248 of the Islands in Bol District received 100% visit to all the settlements. In 2018 the number rose to 350 settlements covered. On the other hand, while 292 settlements in Bol District had zero visits, the number shrunk to 197 in 2018. The same picture was observed in Mani District as well as other districts covered.

Table 1 shows several settlements visited in each of the eight districts over the six-round immunization campaign in 2018. Over 1000 settlements were cumulatively visited in the eight settlements within each round of the campaign with an excess of 100,000 children vaccinated during each round. In the first round, using the GIS tracking system, 124,833 children aged 0-10 years were vaccinated. This increased to 139,777 during the second round and 139,241 in the third round. The trend continued through the sixth round.

In terms of zero dosed children, there was no steady trend, unlike the consistent rise in the number of children vaccinated. However, Table 1 shows that for the first two years there was a decline in the number of zero dosed children from 719 to 409 for the first and second rounds. A slight increase was on the downward for the first and second and third rounds with 719 and 409 respectively. During the third and fourth rounds, there were some increases to 498 and 1,185 respectively. These were largely due to the discovery of more settlements given more precise GIS maps. Subsequently, the trend in the number of zero dosed children started a steady decline.

**Figure 6:**
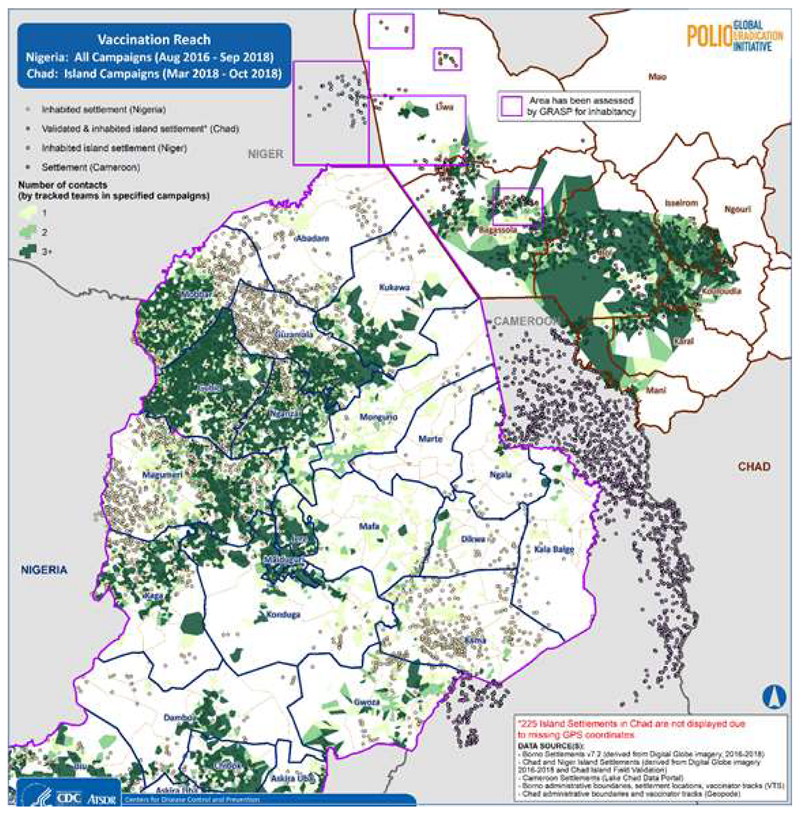
Inhabited Settlements and Vaccination Reach

The map in Fig 6 depicts the number of contacts that a given settlement in Borno State Nigeria and the islands of Chad has received. Contact occurs when a settlement is visited by a tracked vaccination team at least once during a campaign round. Actual contacts were made more than once as shown by the areas in darker shades of green as shown on the map. Particularly for the chad islands, 39% of the inhabited settlement were reached less than 3 times with 18.2% of the population also reached less than 3 times (Table 2). This is largely due to known security challenges within Liwa district in the LAC region of chad where there were attacks prior to some of the campaigns which led to the population relocating.

## Discussion

The project has shown that systematic tracking of vaccination teams during polio campaigns resulted in improved geographic coverage particularly in the Island settlements in Chad. The tracking system ensures critical feedback on settlements reached or completely missed, which enabled supervisors to evaluate performance and missed settlements to be revisited in real-time in the form of mop- up vaccinations. Furthermore, monitoring the movements of vaccination teams using the GIS-enabled tracking system made vaccinators more accountable throughout the campaigns. Children were thus reached, and settlements visited irrespective of size, location, and distance.

Similar results were recorded in the other projects where GIS tracking was deployed, thus making GIS tracking of vaccination teams an important monitoring tool for public health service delivery. In Nigeria, GIS tracking improved immunization coverage for the Nigerian polio program^14^. The effectiveness of immunization is best when a larger proportion of the population is protected against the disease by vaccination, through herd immunity. The existence of a reservoir of the virus and pockets of vulnerable populations, such as chronically missed settlements, and zero dosed children that transmission of poliovirus will continue uninhibited.

Before the deployment of the GIS tracking of vaccinators in Chad, many settlements were missed, and many zero dosed children were found in the settlement that was later uncovered through the GIS mapping. With the GIS system, there were significant reductions in the number of missed and chronically missed settlements for many districts. However, the high numbers of missed settlements in the neighboring countries of Nigeria, Niger, and Cameroon which are attributable mainly to compromised security still pose threats that must be addressed. As some settlements were not accessible to vaccination teams during SIAs, owing to safety concerns, the unvaccinated children will continue to be a source of transmission.

It is relevant to reiterate here, that the outputs from vaccination tracking enhanced the implementation of a number of activities in the tracked sub-districts that were relevant for polio eradication. These included the use of smartphones and tablets to locate chronically missed settlements and unnamed hamlet areas, to review micro plans and align the list of settlements in the GIS database with the ward level micro plan settlement list, and to deploy other interventions in settlements that were missed and inaccessible owing to security concerns during SIAs as per the tracking results^14^. Given the visualization capability of the tracking system, supervisors used the coverage maps alongside the various automated reports to determine whether mop-up immunization activities were effective.

It is important at this point to highlight some of the limitations in the use of the GIS system, as is the case with innovations generally^19^. Although the tracking provides important feedback on the settlements reached by the various vaccination teams using the different reports, recording and analyzing geographic coverage does not translate into actual vaccination of children in the settlements reached^14^. There is still the need to resolve other coverage challenges linked to a poor recording of coverage, a high number of missed children due to absenteeism and non-compliance as well as team attitude. Precise coverage challenges identified in the course of this activity ranged from operational to technical in nature. Operational challenges revolved largely around the loss or broken mobile phones and solar charges device in some health areas which prevented the synchronization of the tracks in those locations. This subsequently impacted on the coverage information from these locations.

Some of the technical challenges were tracks not seen on the server at the end of the campaign days which was due to poor network and in some cases no internet connection, thus delaying tracks submission to the server until the availability of a better network. Also, difficulty in identifying and harmonizing true island settlements for vaccination created a huge disparity. Insecurity challenges was another challenge that led to abandonment of island settlements, low visitation and poor coverage in some of the districts. However, as part of lessons learnt the independent validation of island settlements exercise carried out to harmonize all the island settlements sources in the districts after the second island vaccination had an improved impact in subsequent vaccination.

The determination of whether or not a settlement should be classified as reached was based on the number of GPS tracking points that intersected with the boundaries of the settlement. The concept of vaccination reach based on geographic VTS tracking data is at best a proxy for the actual occurrence of vaccination. If an inhabited place was visited while teams were being tracked, but complete vaccination did not take place, it may lead to an erroneous conclusion that virtually all of the u5 children in that settlement were vaccinated^22^.

Despite these limitations, the use of GIS tracking in conjunction with other innovations has contributed to the successful coverage of previously unvaccinated settlements. Routine tracking of vaccination teams was effective in ensuring that teams reached the settlements they were supposed to cover and enabling supervisors to monitor the level of coverage.

In conclusion, the use of GIS in the polio eradication program in Chad has proven to be most invaluable in improving population immunity and reducing the risk of poliovirus transmission in the country. It is a useful analytical tool for incorporation into health programs, especially in microplanning for polio campaigns, routine immunization, disease surveillance, and response systems^14^. The technology when integrated with local insight can make a significant contribution towards identifying the underserved and unreached settlements for immunization activities and providing proof of coverage, and, thereby, towards more impactful health interventions^20^.

## Figures and Tables

**Table 1:** Number of Islands Tracked between 2017 and 2018 in Chad Mapped by Geographic Information System Technology

REGIONS	DISTRICTS	# of settlements reached							Vaccinated: 0-10 years							zero dose vaccinated: 0-10 years						
		Feb pilot	1^st^ RD	2^nd^ RD	3^rd^ RD	4^th^ RD	5^th^ RD	6^th^ RD	Feb pilot	1^st^ RD	2^nd^ RD	3^rd^ RD	4^th^ RD	5^th^ RD	6^th^ RD	Feb pilot	1^st^ RD	2^nd^ RD	3^rd^ RD	4^th^ RD	5^th^ RD	6^th^ RD
LAC	BAGA SOLA	173	219	238	255	221	212	216	24,420	24,829	26,917	28,474	26,314	26,786	25,949	962	120	76	106	128	101	81
	BOL	83	449	461	483	307	295	316	15,618	53,781	58,109	53,238	39,216	45,539	46,183	6	101	70	83	225	185	48
	ISSEIROM	53	155	155	155	210	231	238	8,590	14,475	15,074	15,539	17,990	19,144	19,115	75	132	64	85	435	253	224
	KOULOUDIA	192	137	146	181	210	219	231	9,482	7,075	8,044	9,439	11,912	11,668	11,784	104	41	28	33	64	33	21
	LIWA	19	55	65	72	84	100	89	1,860	9,672	11,722	12,897	14,976	16,845	15,322	28	163	23	87	111	98	31
	NGOURI	10	18	28	28	32	38	35	887	1,711	4,011	4,145	4,120	4,256	4,269	10	32	66	27	48	34	36
HADJER LAMIS	MANI	16	24	21	23	29	30	30	1,274	4,236	4,305	4,738	3,296	3,606	3,750	1	24	20	22	24	33	31
	KARAL	41	41	42	42	49	53	70	190	9,054	11,595	10,771	9,243	9,216	12,649	4	106	62	55	150	187	66
TOTAL		587	1,098	1,156	1,239	1,142	1,178	1,225	62,321	124,833	139,777	139,241	127,067	137,060	139,021	1,190	719	409	498	1,185	924	538

•   Most zero dose children are 0-11 months

•   During the February pilot activity, Vaccinator Tracking System (VTS) was not used

•   The campaign conducted in 8 Districts housing Island settlements

**Table 2:** Settlements and Populations not reached at least 3 times

Area	Total settlements included in SIAs tracked	Total settlements reached *fewer than* 3 times	Percent settlements reached *fewer than* 3 times	TOTAL population included in SIAs tracked	TOTAL population reached *fewer than* 3 times	Percent population reached *fewer than* 3 times
Borno State, Nigeria	10,717 inhabited (14,782 total)	5,411 inhabited (9,199 total)	**50.5%** of inhabited (62.2% of total)	5,387,967 (VTS/GRASP+IOM)	823,248	**153%**
Chad Islands *	1,341 inhabited (1,431 total)	523 inhabited (612 total)	**39%** of inhabited (42.8% of total)	311,548(ORNL2016 + GRASP)	56,772	**18.2%**
Time Period Covered in Table: August 2016 - September 2018 in **Borno** (*RIC, RES, and H2H included on map*) March 2018-October 2018 in the **Chad Islands**.						

* The number of settlements is based on the available geodatabase of settlements, not Polio microplan settlements. This analysis also excludes population reached through profiling and RIC without GIS evidence
